# Dynamic Mechanical Properties and Damage Evolution Characteristics of Beishan Deep Granite under Medium and High Strain Rates

**DOI:** 10.3390/ma16155235

**Published:** 2023-07-26

**Authors:** Hui Lu, Yue Pan, Kang He, Fei Wang, Lei Gao, Shikun Pu, Erbing Li

**Affiliations:** 1 College of Field Engineering, Army Engineering University of PLA, Nanjing 210007, China; luh007hero@163.com; 2 College of Transportation Engineering, Tongji University, Shanghai 200092, China; 2010782@tongji.edu.cn; 3 College of Defense Engineering, Army Engineering University of PLA, Nanjing 210007, China; 18374984570@163.com (K.H.); wangfei.2017@tsinghua.org.cn (F.W.); gaolei@njust.edu.cn (L.G.); kurtbush@126.com (S.P.)

**Keywords:** Beishan deep granite, medium and high strain rates, SHPB, energy dissipation, damage evolution mechanism

## Abstract

To study the dynamic mechanical properties and damage evolution mechanism of Beishan deep granite under medium and high strain rates, dynamic mechanical tests for the deep granite specimens with different strain rates were conducted using the split Hopkinson pressure bar (SHPB) device. The improved Zhu–Wang–ang (ZWT) dynamic constitutive model was established, and the relationship between strain rate and strain energy was investigated. The test results show that the strain rate in the dynamic load test is closer to the strain rate in the rock blasting state when the uniaxial SHPB test is applied to the granite specimens in a low ground stress state. Peak stress has a linear correlation with strain rate, and the dynamic deformation modulus of the Beishan granite is 152.58 GPa. The dissipation energy per unit volume and the energy ratio increase along with the strain rate, whereas the dissipation energy per unit volume increases exponentially along with the strain rate. There is a consistent relationship between the damage degree of granite specimens and the dissipation energy per unit volume, which correspond to one another, but there is no one-to-one correspondence between the damage degree of granite specimens and the strain rate. To consider the damage and obtain the damage discount factor for the principal structure model, the principal structure of the element combination model was improved and simplified using the ZWT dynamic constitutive model. The change of damage parameters with strain rate and strain was obtained, and the dynamic damage evolution equation of Beishan granite was established by considering the damage threshold.

## 1. Introduction

**Figure 1:**
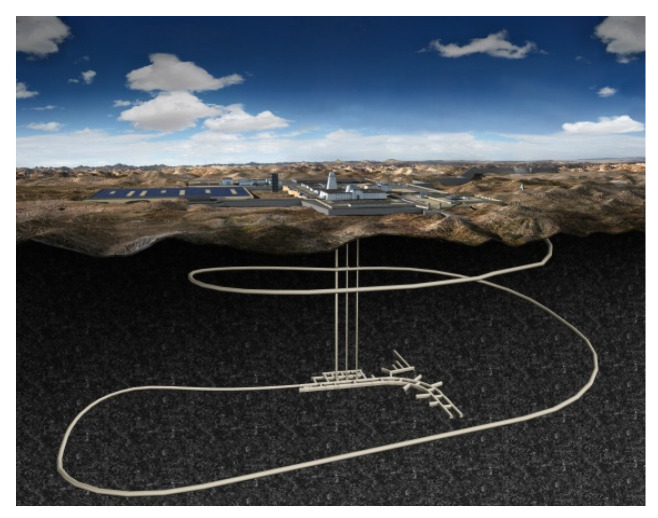
Preliminary picture of the Beishan URL with one access ramp, three shafts, and experimental tunnels.

Deep geological disposal is internationally recognized as the safest and most reliable way to dispose of high-level radioactive waste (HLW). Granite has been chosen as the type of surrounding rock for the geological disposal repository of HLW in China. As a preliminary research project, the Beishan underground research laboratory (URL) (Figure 1) located in Yumen City, Gansu Province, PR China [1], is now at the excavation and construction stage. In the construction process of the URL, the ramp is excavated using a tunnel boring machine, and three shafts and experimental tunnels are excavated by drilling and blasting method. Therefore, the deep rock is inevitably subjected to dynamic loading, and studying the dynamic mechanical properties of Beishan deep granite under the action of medium and high strain rates is crucial to the stability and safety of the URL.

**Figure 2:**
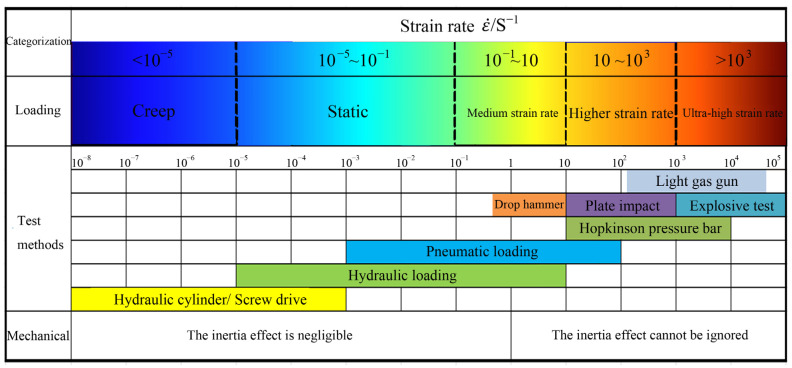
Classification of strain rate-related experimental techniques and mechanical problems of rock.

The strain rate index can measure the response state of materials under a load. Rocks generally bear medium and high strain rates under dynamic loading (Figure 2) [2,3,4]. The strain rate of the rock surrounding the Beishan URL was estimated to be in the range of 10 to 104 s^−1^ under drilling and blasting excavation conditions [5]. Under these conditions, the main testing methods are the drop hammer method, Hopkinson pressure bar, and light gas gun. Various dynamic test methods for rock were compared, the SHPB test was found to be most consistent with the drilling and blasting excavation conditions, and the relevant equipment was frequently used in the field of rock dynamics. A series of conventional dynamic uniaxial compressive tests and coupled static dynamic loading tests were performed using the SHPB system to explore the variable dynamic mechanical behavior and fracture characteristics of medium siltstone at a microscopic scale in the laboratory [6]. Dynamic tests were carried out on granite specimens using a modified SHPB to study the dynamic characteristics and energy evolution of the rock under coupled static–cyclic impact loading and it was concluded that both the dynamic characteristics and the energy evolution of granite are sensitive to the number of repeated impacts and the confining pressure [7]. Using the image processing technique, a new calculation method for crack propagation velocity was proposed, and then the stress–strain characteristics and crack propagation features were analyzed [8].

A comprehensive description of the detailed principles of dynamic NSCB-SHPB technology was refined and applied [9]. The high strain rate characterization of sandstone is performed for two different diameters and five different slenderness ratios of sandstone specimens, and the appropriate specimen size of sandstone for SHPB testing is proposed by checking the strength gain of the rock and the amount of energy absorbed during the tests [10]. To accurately identify and quantitatively calculate the surface cracks of rock mass under SHPB impact loading, an automatic crack detection algorithm was proposed and evaluated by the experiment. The crack identification algorithm proposed can provide a more accurate quantitative method for rock damage by cracks on the rock surface [11]. Dynamic behavior was observed at the side face of the bar specimen using a high-speed video camera, and the captured images were used to analyze surface displacement behavior using a digital image correlation technique [12]. The strain rate was unified in the use of SHPB tests, combined with numerical simulation to analyze the relationship between dynamic strength and freeze–thaw cycles [13]. Based on SHPB laboratory tests, the dynamic mechanical properties and failure mode of sandstone were analyzed, and an SHPB numerical model was established using a particle flow code [14]. The effects of different pulse types and pulse waveforms on the incident waveform and dynamic response characteristics of the specimens were discussed based on the particle flow code. The study determines the critical interval of the dynamic strength of the rock, in which the dynamic strength of the specimen is independent of the strain rate but increases with the amplitude of the incident stress wave [15]. The freeze–thaw damage to granite under impact loading was studied using SHPB tests and numerical simulations [16]. To systematically reveal the dynamic fracture mechanisms of Beishan granite specimens, the key dynamic fracture parameters, including the dynamic fracture toughness, the evolution of dynamic stress intensity factor during fracturing, and the dynamic fracture energy, were obtained [17]. The study of freeze–thaw samples in four different states, i.e., saturated and frozen, saturated and ambient, dry and frozen, and dry and ambient, to investigate the mechanical properties and energy dissipation patterns of surrounding rock under low-velocity cyclic impact is relevant to the damage patterns of rock in weak impact environments [18]. A 50 mm diameter SHPB testing device was used for cyclic impact testing to investigate the mechanical response and energy dissipation characteristics of granite under low-velocity cyclic loading [19].

For various types of rocks, many scholars have conducted a large number of SHPB tests. Beishan granite, as the Beishan URL rock mass, has been the subject of most of the SHPB tests for the study of rock dynamic mechanical properties, without an in-depth study of its dynamic damage evolution and damage constitutive model. The authors refer to the domestic and international experience of rock dynamic tests with medium and high strain rates and use the uniaxial SHPB device to carry out rock dynamic experimental research in order to explore the dynamic damage evolution law of Beishan deep granite. The findings can guide Beishan URL drilling and blasting design and construction.

## 2. Experimental

### 2.1. Test Apparatus

**Figure 3:**
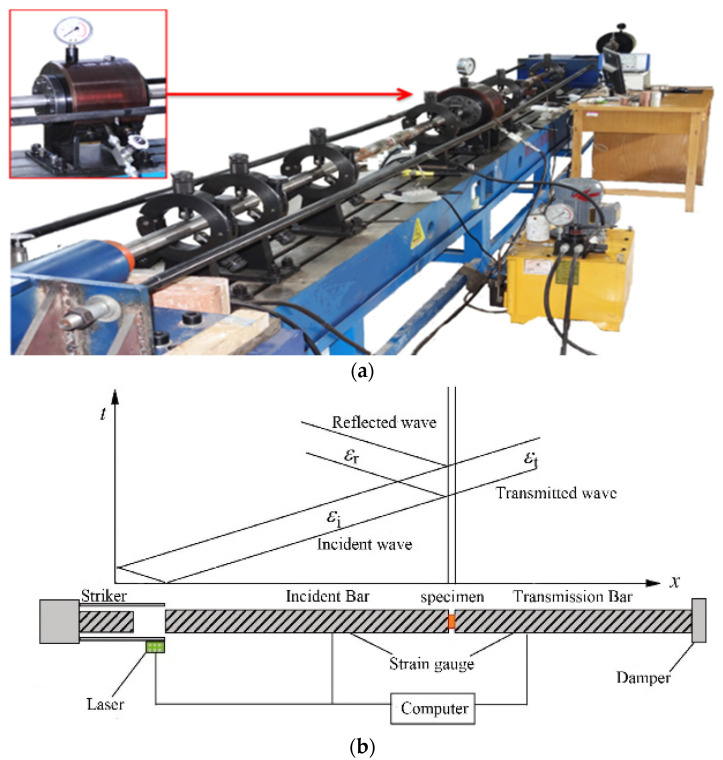
The 50 mm split Hopkinson pressure bar apparatus:(**a**) overall view; (**b**) diagrammatic sketch.

The dynamic mechanical test for the Beishan deep granite was carried out on a test system with the SHPB device (Liwei Techology Co., LTD, Luoyang, China) (Figure 3). The SHPB device consists of five major parts—bar system, axial pressure system, confining pressure system, loading system, and data collection system—which can apply static axial pressure and circumferential pressure to the rock specimens before the dynamic testing. The bar system as the core component of the test device consists of a 3 m incident bar, a 3 m transmitted bar, and a 1 m absorption bar. The bar material is high-strength chrome alloy steel with an elastic modulus of 211 GPa. The ultrasonic wave speed of the bar material is 5201.1 m/s. The hydraulic machine can provide 0–60 MPa pressure to the jack and display the pressure in real-time. The strains on the incident and transmitted bars are converted into voltage signals through strain gauges via the half-bridge box, and the voltage signal is amplified by the KD6009 strain amplifier. Analog voltage signals are then recorded by the super dynamic acquisition instrument, and the strains are recorded and analyzed by the testing analysis software. The velocity measurement device at the end of a firing cavity triggers the acquisition system to collect the operation when it monitors the bullet being fired out of the cavity. A strain gauge (1000-3 AA strain gauge) with a resistance value of 1000 Ω and a sensitivity factor K = 2.00 was selected for this test. The signal test collector sampling frequency is set at 1 M.

### 2.2. Test Procedures

**Figure 4:**
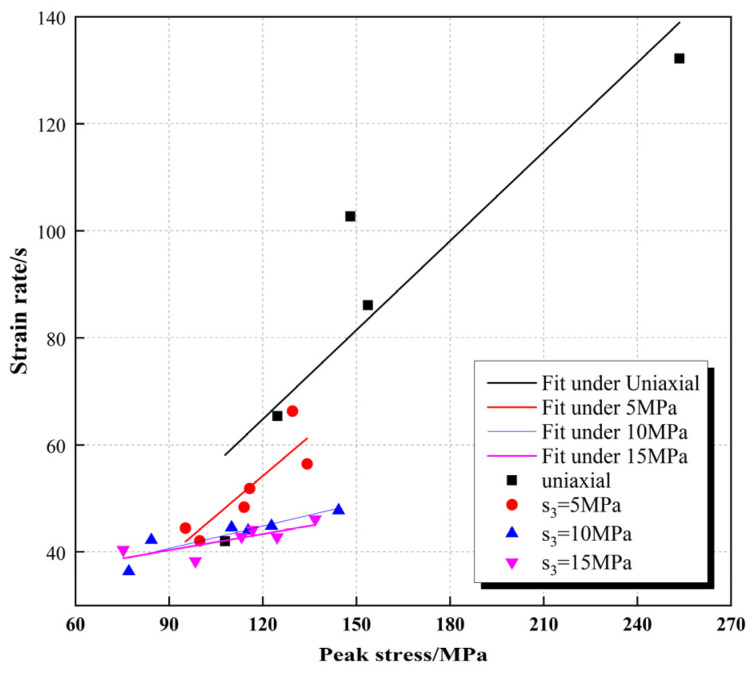
Correlation between strain rate and peak stress subjected to different working conditions.

The uniaxial and triaxial SHPB tests with four circumferential pressures of 0 MPa, 5 MPa, 10 MPa, and 15 MPa were carried out in advance to closely match real working conditions: the uniaxial test achieved high strain rates for a wide range of loading from 42.0 s^−1^ to 132.2 s^−1^, and the failure modes of the specimens were widely distributed from slight damage to high crushing; the highest strain rates were 66.3 s^−1^, 47.7 s^−1^, and 46.1 s^−1^ under surrounding pressures of 5 MPa, 10 MPa, and 15 MPa, respectively (Figure 4). The triaxial lateral restraint improved the stiffness of the specimens, and the maximum achievable strain rate of the equipment was negatively correlated with the magnitude of the surrounding pressure. The specimens did reach the rupture state during the testing. Beishan granite has a low ground stress state [20]. The uniaxial SHPB test has the most applicability in simulating the dynamic loading of drill and blast excavation when the ground stress effect is neglected. Thus, the final decision was made to use uniaxial SHPB to carry out 18 tests loaded with different strain rates. The stress and strain-time curves of the specimens were calculated using the “three-wave method” [21].

### 2.3. Specimen Preparation

The sample used in this study was obtained from Beishan borehole BS06, at a depth of 550–552 m. The laboratory is located in a 22 km long, 7 km wide granite rock mass. The lithology of the rock mass is early gneissic granite and late massive granite. The rocks are linearly distributed in the east–west direction, and the rock types from south to north are gneissic diorite, massive amphibolite, massive diorite, and gneissic granitic amphibolite. Some quartz diorite and tonalite are also interspersed in the late massive granite. The cores are silvery white in appearance, and the Rock Quality Designation is as high as 97.6%. Cylindrical specimens of Φ50 × 25 mm, having a length-to-diameter ratio of 0.5:1, were prepared according to the values recommended by and the accuracy requirements of the International Society of Rock Mechanics [22], the longitudinal wave velocity of each sample was measured using the HS-YS4A rock acoustic parameter tester (Ultrasonic frequency 20 MHZ), and the specimens having abnormal wave velocities were rejected.

**Figure 5:**
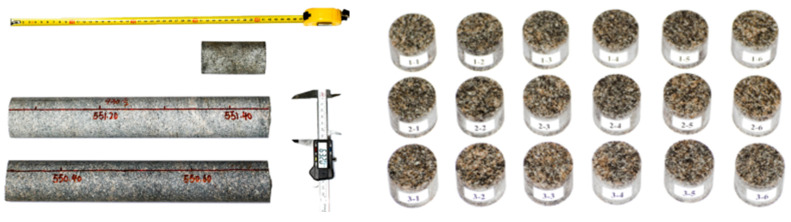
BS06 Sample core and sample preparation.

The specimens were grouped and numbered for dynamic mechanical testing at different strain rates. Some of the specimens that were selected are shown in Figure 5, and the physical properties of each specimen are shown in Table 1.

## 3. Dynamic Characterization

**Figure 6:**
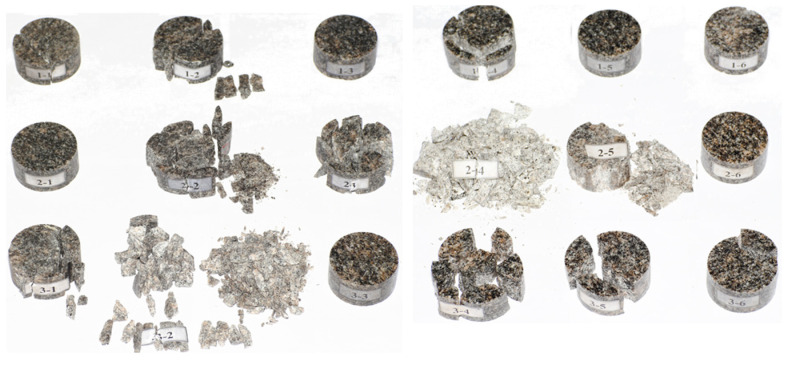
Specimen results.

**Figure 7:**
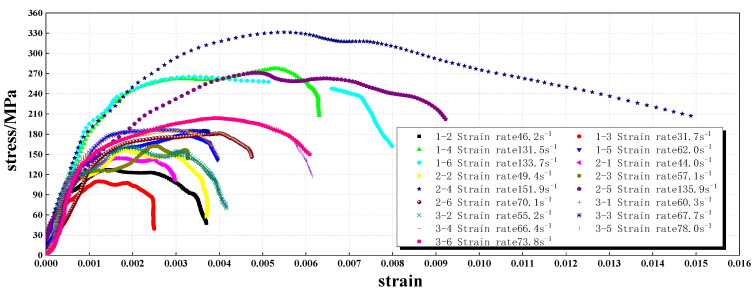
Stress–strain curves of granite subjected to various strain rates.

The air pressure gradient was set to a reasonable value to realize the full damage pattern of specimens, from slight cracking to rupture, and then to crushing and even complete smashing. The test strain rate was widely and uniformly distributed over two ranges (31.7–78.0 s^−1^ and 131.5~151.9 s^−1^). The test result and data processing are shown in Figure 6 and Figure 7.

### 3.1. Damage Analysis

To investigate the damage caused by dynamic loading on the specimens, this study used the longitudinal wave velocities before and after the tests to characterize the damage parameter *D′*:

(1) $$D^{\prime }=1-{\left(\frac{{\overset{-}{V}}_{P}}{V_{P}}\right)}^{2}$$

where $V_{P}$ represents the longitudinal wave velocity of the intact rock specimen before the test, and $\bar{V_{P}}$ represents the mean longitudinal wave velocity of the damaged rock specimen after the test.

The results of the collation test are shown in Table 2. Where through-cracks were broken, the ultrasonic wave velocity of the specimens could not be measured, and the crushed form of the 2–4 specimen was considered to have been destroyed, i.e., the damage parameter *D′* was equal to 1. It was roughly estimated that the use of ultrasonic velocimetry could assess the damage *D′* in the range of 0–0.3.

**Figure 8:**
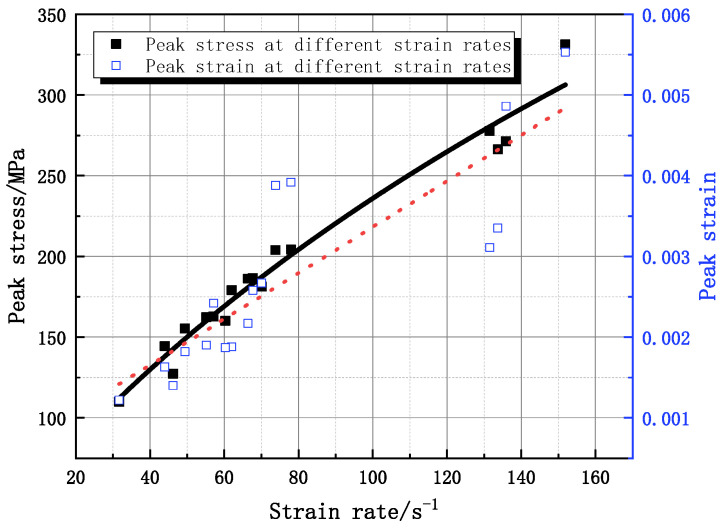
Relationship curves between peak stress and peak strain with strain rate.

The peak stress $\sigma_{peak}$ of the dynamic compressive test shows an obvious linear relationship with the strain rate. The dynamic strength of granite grows approximately linearly with strain rate over a certain strain rate growth range. The increase in dynamic strength slows down at a strain rate above 130 s^−1^ (Figure 8). The peak strain reflects the specimen damage to some extent. Compared with the peak stress–strain rate relationship, the peak strain is more discrete, but in general, the peak strain–strain rate still has a certain linear correlation.

**Figure 9:**
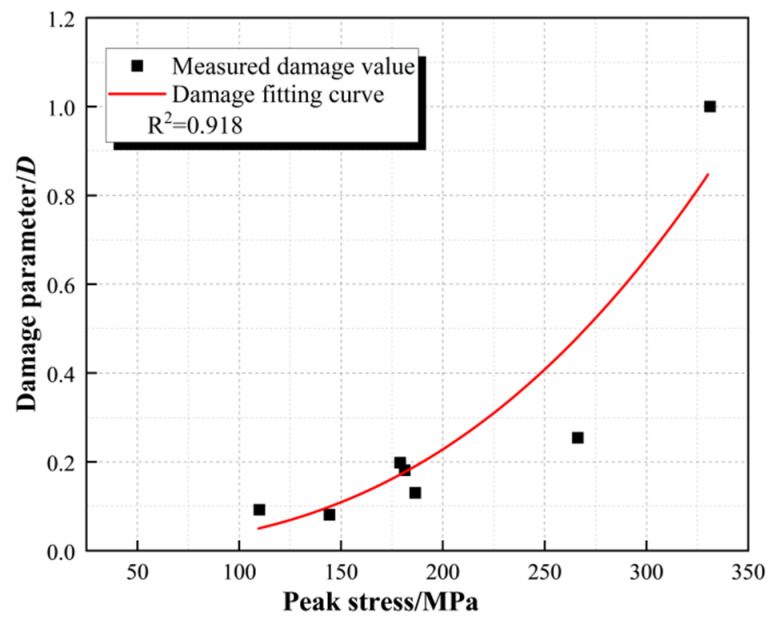
The curve describes the relationship between damage parameters with peak stress.

The relationship between the variation in the damage parameter *D′* and the peak stress characterized by the longitudinal wave velocity was established (Figure 9), and the test results were fitted using the exponential function shown in Equation (2):

(2) $$D\prime =0.02522\exp\left(\frac{\sigma_{peak}}{89.66528}\right)-0.0659$$

When the damage parameter *D′* is 0, so that the specimen is in the critical state of damage, it is brought into Equation (2) to find that the peak stress threshold of dynamic load generated damage is 86.12 MPa; the stress threshold is brought into the fitting curve of peak stress, peak strain, and strain rate in Figure 8, the strain rate threshold is calculated as 20.36 s^−1^, and the strain threshold is 6.31 × 10^−4^.

### 3.2. Dynamic Deformation Modulus Analysis

**Figure 10:**
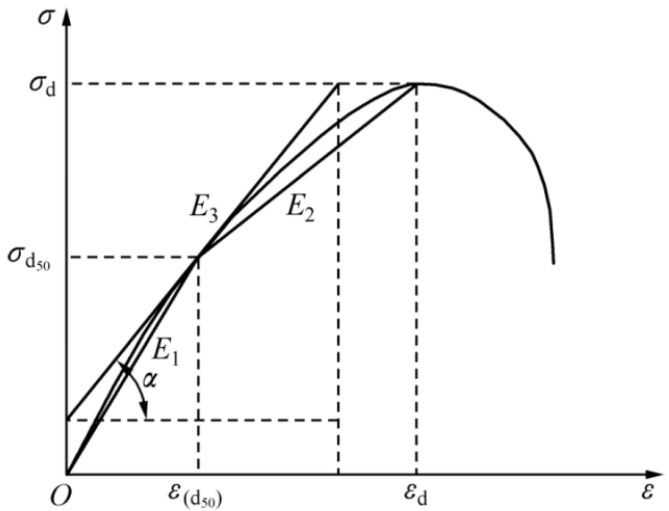
The schematic diagram for defining the dynamic deformation modulus of rock.

The calculation of deformation modulus from rock stress–strain curves is an important method for studying the compressive deformation properties of rocks. There are no definitive norms at home or abroad for determining the deformation modulus from the dynamic stress–strain curves of rocks [23]. Researchers have defined a second secant modulus, loaded section deformation modulus (the slope of the tangent at 50% of the peak stress), and so on. [24,25]. The dynamic stress–strain curve is different from the static load stress–strain curve in that it has no obvious straight line segment, and the error in determining the deformation modulus of the dynamic stress–strain curve regarding the principle of determining the cut line modulus of the static load stress–strain curve is large. To represent the deformation characteristics of the rock over the entire dynamic loading stage and to reduce the error and dispersion, the average of the cut line modulus, the second secant modulus, and the loading section deformation modulus are defined as the dynamic deformation modulus [26]. A schematic diagram of the defined dynamic deformation modulus of the rock ($E_{d}$) is shown in Figure 10, and its equation is given in Equation (3).

(3) $$E_{d}=\frac{1}{3}\left(E_{1}+E_{2}+E_{3}\right)=\frac{1}{3}\left(\frac{\sigma_{d_{50}}}{\varepsilon_{d_{50}}}+\frac{\sigma_{d}-\sigma_{d_{50}}}{\varepsilon_{d}-\varepsilon_{d_{50}}}+\tan\alpha \right)$$

where $E_{1}$, $E_{2}$, $E_{3}$, and $E_{d}$ represent cut line modulus, second secant modulus, loading section deformation modulus, and dynamic deformation modulus, respectively; $\sigma_{d}$ and $\sigma_{d_{50}}$ represent the peak stress and half of the peak stress; $\varepsilon_{d}$ and $E_{d50}$ represent the peak stress and the strain corresponding to 50% of the peak stress, respectively; α is the angle between the tangent line at 50% of the peak stress and the epsilon axis.

**Figure 11:**
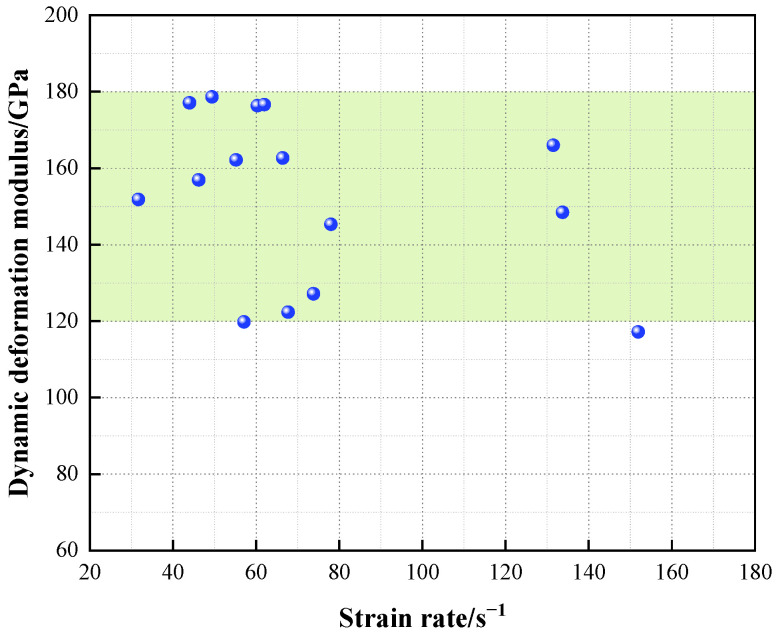
Dynamic deformation modulus of granite at different strain rates.

The magnitude of the granite dynamic deformation modulus, like the static deformation modulus [27], is related to the structure of the rock itself and does not vary with the strain rate. The dynamic deformation modulus of granite under different strain rates can be solved by using the formula for the power average dynamic deformation modulus (Figure 11). The deformation modulus of Beishan granite is in the range 120–180 GPa and has a mean value of 152.58 GPa; the discrete variation of the deformation modulus is still mainly related to factors such as specimen processing accuracy.

### 3.3. Energy Damage Evolution Analysis

In the granite test that used the SHPB device, the energy carried by the incident, reflected, and transmitted waves from the beginning of loading to unloading was calculated using Equations (4)–(6):

(4) $$W_{I}\left(t\right)=EAc\int_{0}^{t}\varepsilon_{I}^{2}\left(t\right)dt$$

(5) $$W_{R}\left(t\right)=EAc\int_{0}^{t}\varepsilon_{R}^{2}\left(t\right)dt$$

(6) $$W_{T}\left(t\right)=EAc\int_{0}^{t}\varepsilon_{T}^{2}\left(t\right)dt$$

where *E*, *A*, and *c* represent the modulus of elasticity, cross-sectional area, and stress wave propagation velocity in the rod, respectively; $\varepsilon_{I}$, $\varepsilon_{R}$, and $\varepsilon_{T}$ represent the strains of the incident, reflected, and transmitted bar, respectively; and $W_{I}\left(t\right)$, $W_{R}\left(t\right)$, and $W_{T}\left(t\right)$ represent the incident energy, reflected energy, and transmitted energy, respectively.

The dissipated energy $W_{s}\left(t\right)$ of specimens during the SHPB test was calculated according to the principle of energy conservation:

(7) $$W_{S}\left(t\right)=W_{I}\left(t\right)-W_{R}\left(t\right)-W_{T}\left(t\right)$$

According to the results for the kinetic energy of specimen fragments during the SHPB test of Zhang et al. [28], it was observed that the energy dissipated by the rock was mainly used in the formation of cracks, the extension of cracks, and the growth of cracks, but the kinetic energy of fragments $W_{K}\left(t\right)$ and other dissipated energy, such as heat and radiation energy, was neglected. Cai et al. [29] found that the results for granite showed that the lateral inertia effect had less influence on the test results when the strain rate was below a certain value.

To reasonably compare the magnitude of the total dissipated energy, the total dissipation energy was converted to dissipation energy per unit volume *W*, which is the ratio of total dissipation energy $W_{K}\left(t\right)$ to the volume $V_{s}$ of specimens.

(8) $$W=\frac{W_{S}}{V_{S}}$$

The strength of the energy dissipation of the specimens under the dynamic impact of SHPB can be characterized using the energy dissipation ratio *η*, which is the ratio of dissipated energy to incident energy.

(9) $$\eta =\frac{W_{S}}{W_{I}}$$

**Figure 12:**
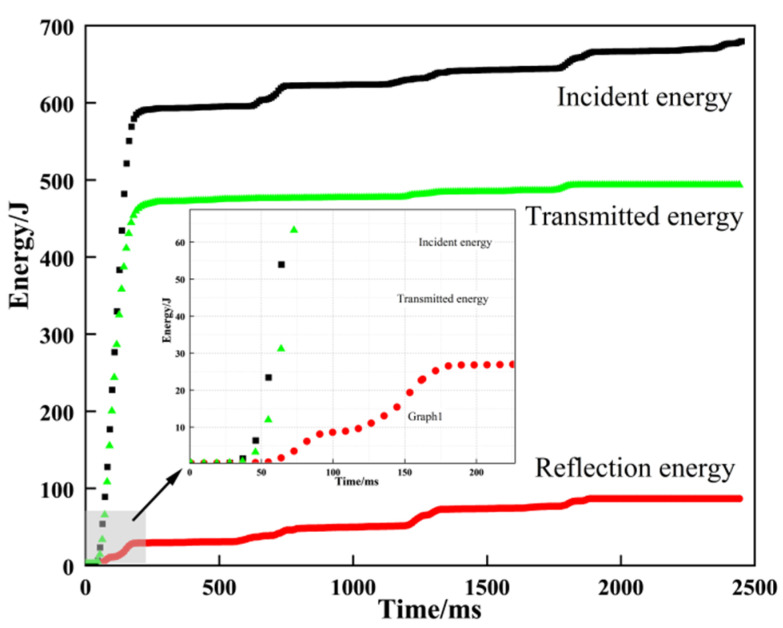
Curves describing the relationship between energy and test time during the SHPB test.

**Figure 13:**
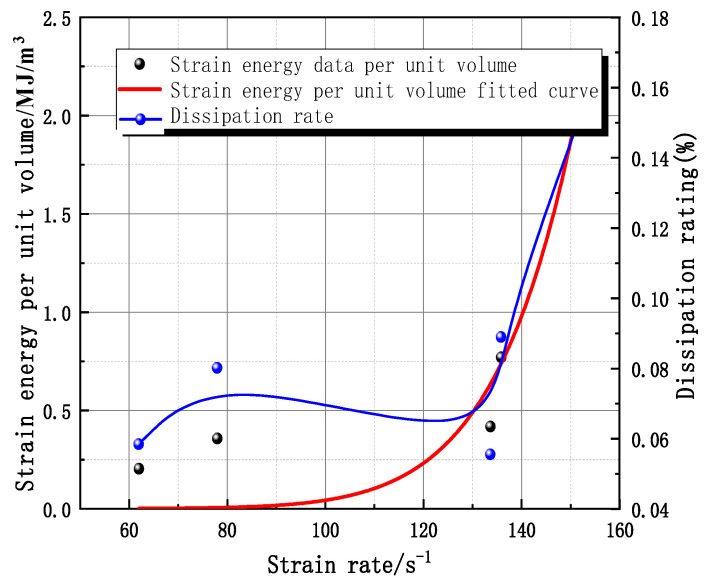
Curves describing the relationship between dissipation energy per unit volume and dissipation rate with strain rate.

Figure 12 shows the relationship between the magnitude of the incident, transmitted, and reflected energy with time for granite in the SHPB test. With the increase in time, the incident energy, transmitted energy, and reflected energy increase accordingly and stabilize after they reach a certain value. The energy carried by the incident wave is greater than the sum of the energy carried by the reflected wave and the transmitted wave, and the difference between the energy of the incident wave minus the energy carried by the reflected wave and the transmitted wave is the energy absorbed by the granite during the impact. The energy carried by the transmitted wave is greater than the energy carried by the reflected wave, which indicates that some energy is transferred to the transmitted bar through the specimen. The dissipation energy per unit volume increases with the strain rate in an exponential relationship ($R^{2}$ = 0.886). The energy dissipation ratio largely increases with an increase in strain rate in a complex nonlinear relationship (Figure 13).

**Figure 14:**
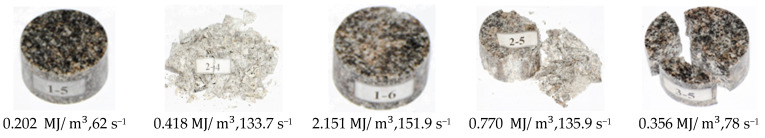
Various degrees of specimen damage.

Figure 14 shows the relationship between the degree of granite damage and the dissipation energy per unit volume with strain rate. It can be seen from the figure that there is a consistent positive relationship between the degree of destruction of the specimens and the dissipation energy per unit volume. When the specimens are broken into blocks, the dissipation energy per unit volume is low. When the core is crushed, the dissipation energy increases accordingly. In particular, when the degree of destruction is coarse grain crushing or complete powder crushing, the dissipation energy per unit volume increases significantly (the degree of destruction of specimen 2–4 develops to specimen 2–5, the increase in strain rate is 16.0 s^−1^, and the increase in dissipation energy per unit volume is 1.381 MJ/${\;m}^{3}$, which far exceeds the percentage increase in the strain rate). This is mainly because, after the specimen is impacted by the incident bar, the energy gained by the specimen is released in the form of microcrack expansion within the material, and thus the number of microcracks generated and developed is related to the dissipation energy per unit volume. When the dissipation energy per unit volume is large, the generation of a large number of microcracks directly leads to crushing damage of the material; when the dissipation energy per unit volume is low, the number of microcracks is relatively smaller and the damaged particles are larger. However, there is no one-to-one correspondence between the degree of specimen damage and the strain rate; at low strain rates, crushing damage is presented, whereas at high strain rates, rupture into blocks is presented (specimens 3–5 had a strain rate of 78 s^−1^, and the rock was broken into blocks, while specimen 1–6 had a strain rate of 133.7 s^−1^, and the rock generally remained whole). It can be seen that the degree of granite damage generally increases with the increase in dissipation energy per unit volume, but not with the increase in strain rate.

## 4. Constitutive Model

### 4.1. ZWT Improvement to Constitutive Model

The mechanical response of granite under dynamic loading usually involves the effect of dynamic strength increase and the strain rate of the material. The magnitude of the strain rate of Beishan granite under SHPB test conditions is a medium strain rate, and the dynamic mechanical effect of granite under this condition is dominated by the effect of the dynamic loading strength increase. For the nonlinear intrinsic model under a high strain rate and large deformation conditions, Sun et al. [30] established a model consisting of two Maxwell bodies and a nonlinear elastomer in parallel, namely the ZWT model. In this model, the two Maxwell bodies reflect the mechanical response under high-strain rate conditions and a low-strain-rate viscoelastic mass, respectively. The Maxwell bodies describing the low-strain-rate viscoelastic response of the material under medium-high strain rate conditions do not have enough time to relax, and in this study they can be reduced to linear springs having modulus *E*_1_:

(10) $$\sigma =f_{e}\left(\varepsilon \right)+E_{1}\varepsilon +E_{2}\int_{0}^{t}\dot{\varepsilon }\left(\tau \right)\exp\left(-\frac{t-\tau }{\varphi_{2}}\right)d\tau$$

(11) $$f_{e}\left(\varepsilon \right)=E_{0}\varepsilon +\alpha \varepsilon^{2}+\beta \varepsilon^{3}$$

where $f_{e}\left(\varepsilon \right)$ represents a nonlinear spring model expressed as a cubic function of *ε* by the three parameters, $E_{0}$, α, and β; $E_{1}$, $E_{2}$, and $\varphi_{2}$ represent the elastic constants and relaxation time of the two Maxwell bodies, respectively.

The results of the SHPB dynamic mechanical compression test in Chapter 3.1 (Figure 7) show that the test strain magnitude does not exceed 10^−2^, and combined with the results of the prior static test on granite, the stress–strain curve has a linear character at this stage. Therefore, the nonlinear spring can be simplified as a linear spring with a modulus of elasticity *E*_0_. The two simplified linear springs are combined, so that $E_{a}=E_{0}+E_{1}$, and the strain rate is kept constant during the tests. The integral term containing $\dot{\varepsilon }$ can be simplified again as follows:

(12) $$\sigma =E_{a}\varepsilon +E_{2}\varphi_{2}\dot{\varepsilon }\left[1-\exp\left(-\frac{\varepsilon }{\dot{\varepsilon }\varphi_{2}}\right)\right]$$

Granite is an elastic-brittle material. With loading, the damage fracture of the specimen increases until the specimen is damaged, and the appearance of damage fracture reduces the strength of the material. According to the strain equivalence assumption by Lemaitre, the strain under real stress is equivalent to the strain under effective stress in the virtual non-destructive state. The intrinsic structural relationship of granite can be expressed as

(13) $$\sigma =\left(1-D\right)\tilde{\sigma }$$

where σ represents the effective stress; $\;\tilde{\sigma }$ represents the real stress; *D* represents the damage variable (i.e., when *D* is 0, it means there is no damage, and when *D* is 1, the material is completely damaged and loses its strength).

The dynamic damage constitutive model of Beishan granite at a constant strain rate can be expressed as:

(14) $$\sigma =\left(1-D\right)\left\{E_{a}\varepsilon +E_{2}\varphi_{2}\dot{\varepsilon }\left[1-\exp\left(-\frac{\varepsilon }{\dot{\varepsilon }\varphi_{2}}\right)\right]\right\}$$

Through an analysis of the damage evolution law, the damage can be related to ε and $\dot{\varepsilon }$, and the damage evolution under dynamic loading can be regarded as a stress-promoted, thermally activated process [31]. Assuming the existence of a strain threshold $\varepsilon_{0}$, no damage occurs when the peak strain is less than the threshold, and when the peak strain is greater than or equal to the threshold, the damage evolution is a thermally activated process, and the dynamic damage evolution equation for granite is:

(15) D=0KDε˙αε−ε0 ε<ε0 ε≥ε0

where $K_{D}$ and α represent the dynamic response parameters of the material. Substituting the damage evolution equation into Equation (14), a dynamic constitutive model of granite that considers the damage evolution can be obtained, and the strain threshold $\varepsilon_{0}$ of Beishan granite, as calculated in Section 3.1 is used.

(16) $$\sigma =\left[1-K_{D}{\dot{\varepsilon }}^{\alpha }\left(\varepsilon -\varepsilon_{0}\right)\right]\left\{E_{a}\varepsilon +E_{2}\varphi_{2}\dot{\varepsilon }\left[1-\exp\left(-\frac{\varepsilon }{\dot{\varepsilon }\varphi_{2}}\right)\right]\right\}$$

### 4.2. Parameter Sensitivity Analysis and Acquisition

**Figure 15:**
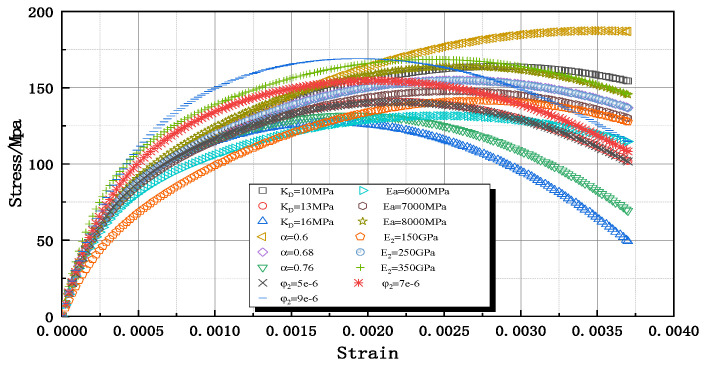
Sensitivity analysis of constitutive parameters for damage.

The dynamic constitutive model of granite that considers damage evolution contains a total of five parameters, $K_{D}$, α, $E_{a}$, $E_{2}$, and $\varphi_{2}$. Because the stress–strain curve has complete loading and unloading for the strain rate $\dot{\varepsilon }$ = 46.2 s^−1^ of granite specimens, the result of 1–2 specimen is used as the benchmark. The effect of each parameter on the present constitutive curve was analyzed by adjusting the value of a single parameter and fixing the other parameters at a constant value (Figure 15).

It can be seen that $K_{D}$ and α, which are the material dynamic response parameters of damage evolution, mainly affect the decay trend of the granite elastic modulus above the strain threshold $6.313\times 10^{-4}$; the larger the $K_{D}$, the faster the rate of damage decay; the larger the α, the smaller the rate of damage decay. The change in $E_{a}$ has a less effect on the slope of the curve onset, whereas $E_{a}$ has a much smaller value than $E_{2}$, which indicates that the simplified elastomer-sharing effect is smaller in the parallel body. $\varphi_{2}$, $E_{a}$, and $E_{2}$ all reflect the effect on the slope of the curve and the peak stress, which together play a role in regulating the shape of the curve. Sensitivity parameters can be calibrated using artificial intelligence methods [32].

**Figure 16:**
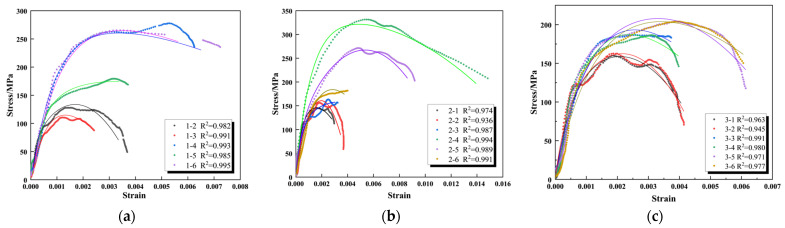
Fitting of constitutive damage to the test: (**a**) specimen 1—X; (**b**) specimen 2—X; (**c**) specimen 3—X.

After the parameter sensitivity analysis was completed, the results of the dynamic SHPB compression test were fitted using the ZWT improved model function (Figure 16). It can be seen that the dynamic intrinsic model of granite which considers damage evolution not only describes the elastic section better but also reflects the trend of the plastic section and shows the same peak intensity as the test. This shows that a combined model of components and damage evolution gives a reasonable physical explanation for the dynamic response of granite, and the intrinsic model reflects the dynamic mechanical properties of granite well. However, at the same time, the model is a physical simplification of a complex material, such as a rock with few parameters, so the model cannot completely fit the undulations in the curve.

Table 3 shows the statistics of the intrinsic fitting parameters for each specimen with the law for increasing strain rate. $K_{D}$ and α decrease with increasing strain rate, which indicates that the rate of damage evolution slows with an increase in strain rate. The parameters *E*_2_ and $\varphi_{2}$ are approximately 200 GPa and 10 μs, respectively, whereas the $E_{a}$ parameter takes small and unstable values. *E*_2_ and $\varphi_{2}$ reflect the dynamic mechanical properties of most of the crystals in the granite, whereas $E_{a}$ reflects that there are small components that can be described as having elastic properties under dynamic loading conditions and do not have a specific relationship with the strain rate. The $E_{a}$ of Beishan granite has reasonable values in the range of 6 × 10^3^ to 1 × 10^5^ MPa. $K_{D}$ and α decrease with an increase in strain rate, which indicates that the rate of granite damage evolution slows down with increasing strain rate.

### 4.3. Damage Evolution Equation

**Figure 17:**
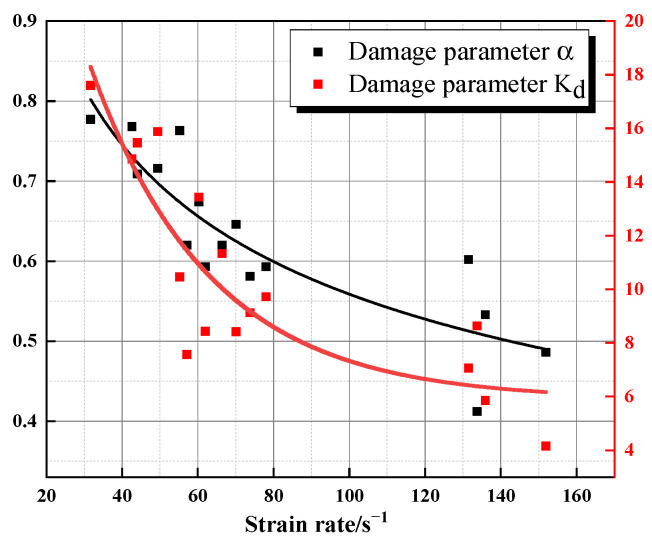
Distribution of damage parameters with strain rate.

With rock loading, rock damage accumulates. This can be understood as the mechanical decay of material during the loading process that is accumulated to the extent that the material fails. Therefore, it is necessary to establish a suitable damage evolution equation for the dynamic constitutive equation that considers granite damage. Figure 17 shows the distribution laws for parameter α and parameter $K_{D}$ with strain rate, respectively. The exponential function and power function are fitted to obtain the following relations, respectively:

(17) $$\alpha =2.3826\times {\dot{\varepsilon }}^{-0.315}$$

(18) $$K_{D}=33.6357\times \exp\left(-\frac{\dot{\varepsilon }}{31.7683}\right)+5.88445$$

**Figure 18:**
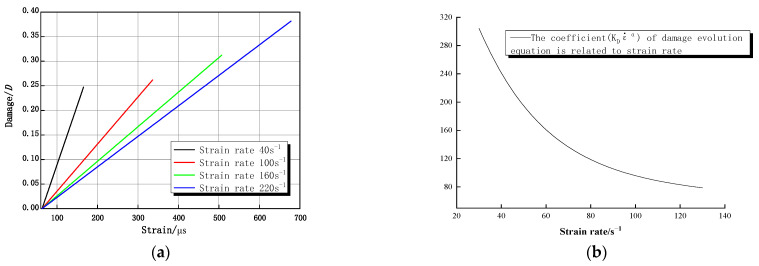
Damage evolution function and its coefficient variation rule: (**a**) damage versus strain curve; (**b**) coefficient curve.

Based on the assumptions presented in Section 3.3, the damage evolution for a constant strain rate loading resembles a thermal activation process after the strain crosses a threshold. Figure 18a depicts the damage development law with strain for each strain rate condition. To better understand the influence of strain rate on damage evolution, $K_{D}{\dot{\varepsilon }}^{\alpha }$ is used as the coefficient for damage evolution of Equation (15). Figure 18b shows that the coefficient in the equation decreases with increasing strain rate, and the higher the strain rate, the slower the rate of damage decrease. At the same time, the higher the strain rate, the greater the strain, and the final cumulative damage increases with increasing strain rate. However, this incremental rate seems to be contrary to our experimental results. This is because the uniaxial SHPB does not have any lateral restraint, and the specimen is damaged when it is loaded with spalling and missing pieces, which results in the loss of the bearing capacity of the rock. At this time, the incident bar continues to apply load under the action of inertia, and the final damage to the specimen is greater than the damage at the peak strain. The damage to the complete rock sample, as measured by ultrasonic wave velocity, is shown in Table 2, and the damage variable *D* is approximately 0.2 at a strain rate of 60 s^−1^, which is closer to the case in Figure 18a.

## 5. Conclusions

In this paper, Beishan deep granite was used as the research object, and its damage mechanism and mechanical response under medium and high strain rates were explored using the SHPB testing device. The main conclusions are as follows:

- The uniaxial SHPB testing device was used to perform dynamic mechanical compression tests on the Beishan deep granite. The damage to the specimens was distributed from slightly cracked to highly crushed, and there was a high correspondence between the crushing degree and the strain rate. By measuring the longitudinal wave velocity before and after the test, the relationship between the damage parameters and the peak stress was found. A strain rate threshold of 20.36 s^−1^ and strain threshold 6.31 × 10^−4^ were obtained for the dynamic loading damage of Beishan granite.
- With the strain rate increases, the compressive strength of the granite tends to increase, and the compressive strength has a linear correlation with the strain rate. The dynamic deformation modulus of the rock is the same as the static deformation modulus, which is related to the structure of the rock itself and does not change with the change in strain rate. The deformation modulus of Beishan granite is in the range of 120 GPa to 180 GPa, with an average value of 152.58 GPa.
- There was an exponential relationship between strain rate and dissipation energy per unit volume; strain rate increased with an increase in energy dissipation ratio in a complex, nonlinear relationship. There was good agreement between the degree of damage to granite specimens and the dissipation energy per unit volume, which corresponded to each other. When the specimen was broken into blocks, the dissipation energy per unit volume was correspondingly less; when the core was crushed, the energy dissipated was correspondingly more. However, there was no one-to-one correspondence observed between the degree of damage of the specimen and the strain rate; crushing damage was presented at a low strain rate, whereas the specimen was broken into blocks at a high strain rate.
- For the dynamic mechanical properties of Beishan granite, the elemental combination intrinsic model that considers damage was obtained by improving and simplifying, based on the ZWT model. The values of the parameters of the present constitutive model of granite in Beishan were obtained by fitting the stress–strain curve. The damage evolution coefficient in the constitutive model was extracted to obtain the relationship between the damage parameters and the strain rate and the strain, and a dynamic damage evolution equation for the granite of Beishan was established by combining the damage threshold.

## Figures and Tables

**Table 1:** Mean values of physical parameters of specimens.

Length mm	Diameter mm	Length-Diameter Ratio	Density (g/cm^3^)	P-Wave Velocity (m/s)	Uniaxial Compressive Strength (MPa)
25.31	49.33	0.51	2.65	5341.12	200

**Table 2:** Pressure dynamic test results (mean values).

No.	Strain Rate (s)	Peak Stress (MPa)	Before-Test Longitudinal Wave Speed (m/s)	Post-Test Longitudinal Wave Speed (m/s)	*D′*	No.	Strain Rate (s^−1^)	Peak Stress (MPa)	Before-Test Longitudinal Wave Speed (m/s)	Post-Test Longitudinal Wave Speed (m/s)	*D′*	No.	Strain Rate (s^−1^)	Peak Stress (MPa)	Before-Test Longitudinal Wave Speed (m/s)	Post-Test Longitudinal Wave Speed (m/s)	*D′*
1-1	Null test					2-1	44.0	144.4	5244.6	5027.7	0.081	3-1	60.3	160.1	5385.11	Rupture	-
1-2	46.2	127.3	5443.0	Rupture	-	2-2	49.4	190.4	5056.0	Rupture	-	3-2	55.2	162.4	5378.72	Rupture	-
1-3	31.7	109.9	5175.8	4932.0	0.092	2-3	57.1	162.8	5560.4	Rupture	-	3-3	67.7	186.5	5391.49	5027.7	0.130
1-4	131.5	277.6	5372.3	Rupture	-	2-4	151.9	331.3	5376.6	Rupture	1	3-4	66.4	186.2	5021.78	Rupture	-
1-5	62.0	179.1	5493.4	4920.4	0.198	2-5	135.9	271.3	5281.3	Rupture	-	3-5	78.0	204.2	5326.32	Rupture	-
1-6	133.7	266.3	5495.6	4747.7	0.254	2-6	70.1	181.3	5404.3	4890.4	0.181	3-6	73.8	203.9	5521.74	Rupture	-

**Table 3:** Damage constitutive model parameter—strain rate.

Strain Rate	K_D_	α	E_a_/GPa	E_2_/GPa	φ_2_/μs
31.7	17.597	0.777	5.957	177.694	29.193
42.6	14.860	0.768	74.426	251.567	5.068
44	15.463	0.709	50.368	210.791	11.855
49.4	15.874	0.716	109.688	54.653	10.434
55.2	10.466	0.763	83.523	195.436	6.128
57.1	7.568	0.620	29.865	207.197	9.356
60.3	13.440	0.674	68.417	170.526	8.638
62	8.434	0.593	33.020	252.545	8.075
66.4	11.333	0.620	35.838	183.111	15.545
70.1	8.417	0.646	20.000	125.550	31.568
73.8	9.129	0.581	39.398	142.640	16.178
78	9.726	0.593	61.659	163.160	9.020
131.5	7.062	0.602	22.192	219.579	9.758
133.7	8.636	0.412	20.000	255.998	7.844
135.9	5.850	0.533	61.043	177.520	4.198
151.9	4.159	0.486	20.674	233.319	8.747

## Data Availability

The data presented in this study are available on request from the corresponding author.
